# The Relationship between Alternative Healthy Diet Index and Cognitive Function in the Older Adults: The Mediating Effect of Depressive Symptoms

**DOI:** 10.3390/nu14142856

**Published:** 2022-07-12

**Authors:** Zhonghai Lu, Chen Chen, Jiesong Zhang, Xueyan Wang, Dongfeng Zhang, Suyun Li

**Affiliations:** Department of Epidemiology and Health Statistics, School of Public Health, Qingdao University, Qingdao 266021, China; 2020021086@qdu.edu.cn (Z.L.); 2020021065@qdu.edu.cn (C.C.); 2021021096@qdu.edu.cn (J.Z.); 2021021081@qdu.edu.cn (X.W.); zhangdongfeng@qdu.edu.cn (D.Z.)

**Keywords:** dietary quality, depressive symptoms, AHEI-2010, cognitive function, NHANES

## Abstract

This study aimed to investigate the association between the alternative healthy eating index-2010 (AHEI-2010) with cognitive function and the mediating role of depressive symptoms in older adults using the data from the 2011–2014 National Health and Nutrition Examination Survey (NHANES). The AHEI-2010 was calculated from NHANES individual food data and Food Patterns Equivalents Database (FPED) diet data. Cognitive function was assessed by the Consortium to Establish a Registry for Alzheimer’s disease (CERAD, memory function indicator), Word Learning sub-test, digital symbol substitution test (DSST, processing speed indicator), animal fluency test (AFT, executive function indicator), and the comprehensive z-score (global cognition indicator). A weighted multiple linear regression model was used to explore the relationship between AHEI-2010 and cognitive function, and Karlson–Holm–Breen (KHB) method was used to explore the mediating effect of depressive symptoms. A total of 2644 participants were included in this study. Participants with higher AHEI-2010 scores were more likely to have higher scores in DSST, AFT, and comprehensive z-score compared with the lowest quartile. Depressive symptoms play a significant mediating role between AHEI-2010 and cognitive function. The proportion of mediating in CERAD, DSST, AFT, and comprehensive z-score was 14.14%, 9.10%, 9.15%, and 10.47%, respectively. This study found that higher dietary quality was associated with better cognitive function. In addition, depressive symptoms may be an important pathway linking diet and cognitive function.

## 1. Introduction

Cognitive decline is a feature of the aging process, which is characterized by the impairment of intellectual abilities such as memory, reasoning, executive function, and information processing speed [1]. There are many reasons for cognitive decline, such as neurodegeneration due to the deposition of extracellular amyloid plaques and hyper-phosphorylation of tau protein, or the increase in inflammatory mechanism and oxidative stress [2,3,4]. Additionally, according to an experimental study, nutrients may play a vital role in these mechanisms of neurodegeneration [5]. It is worth noting that a nutritious diet is an important factor that is easy to change. Additionally, people do not consume nutrients in isolation. Rather, they consume food groups consisting of multiple foods, and there may be synergistic effects of complex food combinations [6]. As a result, more and more scientists focused on dietary patterns or dietary quality to better understand the link between diet and cognitive decline or dementia [7].

The Alternative Healthy Diet Index (AHEI) is a measure of diet quality and represents the extent to which overall healthy diet patterns are followed, which provides better recommendations to improve health risk factors as well as greater predictive power for chronic disease relative to the Healthy Diet Index (HEI) [8]. So far, only one study has been reported on the relationship between AHEI-2010 and cognitive function in American adults, which was conducted on Hispanics [9]. In addition, a study of older adults in China found that a higher AHEI-2010 value in middle age was associated with a lower risk of cognitive impairment in old age [10]. However, the underlying mechanisms between AHEI and cognitive function are unclear. Therefore, exploring the pathways involved and elucidating the intrinsic link between diet and cognitive function is necessary.

Several studies reported that lower dietary quality was associated with a higher risk of depression [11,12,13,14]. At the same time, several studies on the causal relationship between depression and cognitive function have shown that depressive symptoms may be a risk factor for cognitive decline [15,16,17,18]. Thus, people with poor-quality diets may suffer from depression, which in turn can adversely influence cognitive function.

In conclusion, the above evidence suggests that depression may be in the causal chain between diet and cognitive function. However, so far, no study has explored whether depression plays a mediating role in the relationship between dietary quality and cognitive function. Therefore, the purpose of this study was to use the data of the National Health and Nutrition Examination Survey (NHANES) and the Food Patterns Equivalents Database (FPED) diet data to analyze the relationship between AHEI-2010 and cognitive function and further investigate whether this relationship was mediated by depressive symptoms.

## 2. Materials and Methods

### 2.1. Study Population

**Figure 1:**
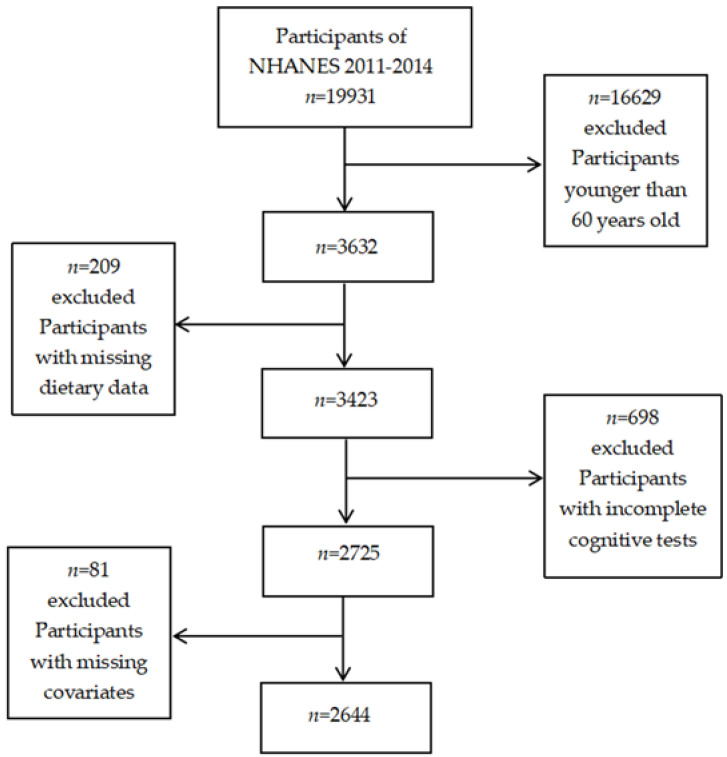
Flow chart for the selection of included sample.

The population in this study was from NHANES (https://www.cdc.gov/nchs/nhanes/index.htm) (access date: 11 July 2022). NHANES, an ongoing biennial cross-sectional survey administered by the Centers for Disease Control and Prevention, aimed to assess the health and nutritional status of adults and children in the United States. Participants were selected through a complex, multistage, probabilistic sampling design, so the sample represents the non-institutionalized U.S. civilian population. In this study, we combined data from two NHANES cycles (NHANES 2011–2012 and 2013–2014), which consist of a series of cognitive function tests. A total of 19,931 participants took part in the survey between 2011 and 2014. Our analysis was limited to adult participants aged 60 and over who participated in the cognitive function survey (*n* = 16,299). Participants with missing dietary data were excluded (*n* = 209). Those who did not complete the cognitive function test (*n* = 698) were also excluded. In addition, participants with missing covariates such as race, education status, smoking, and chronic diseases were also excluded (*n* = 81). Finally, 2644 older adults aged 60 and over were included in the analysis (Figure 1).

### 2.2. AHEI-2010

The AHEI is a priori diet index based on the Healthy Eating Index (HEI), created in 2002. Previous studies showed that the AHEI-2010 had a better predictive ability for chronic disease relative to the HEI [19]. AHEI-2010 consists of 11 components: six recommended components: vegetables, fruits, whole grains, nuts and legumes, omega-3 fatty acids, and polyunsaturated fatty acids (PUFA); one moderate component: alcohol; four components that must be limited or avoided: sugary beverages and fruit juices, red and processed meats, sodium and trans fatty acids. The score of each component ranges from 0 to 10. Add the scores of each component to get the total score of AHEI-2010: 0–110 points. However, trans fats were not provided in the NHANES dietary database. As a result, the highest total score of AHEI-2010 was adjusted from 110 to 100 (excluding the trans fatty acids). NHANES individual food data and Food Patterns Equivalents Database (FPED) diet data were used to estimate food supply to build an AHEI-2010 food group. Each individual food was classified according to the USDA food code. After creating the AHEI food and nutrients, the SAS code was built to calculate the AHEI-2010 score.

### 2.3. Cognitive Function

Cognitive function was assessed by the Consortium to Establish a Registry for Alzheimer’s disease (CERAD) Word Learning subtest, Animal Fluency Test (AFT), and Digital Symbol Substitution Test (DSST). The CERAD assessed immediate and delayed learning ability of new verbal information (memory sub-domain) and consisted of three consecutive learning trials and a delayed recall. The AFT examined categorical verbal fluency, a component of executive function. Participants were asked to name as many animals as possible in one minute. The DSST, a performance module from the Wechsler Adult Intelligence Scale, relied on processing speed, sustained attention, and working memory. Participants had two minutes to match the symbols corresponding to numbers. The score was defined as the total number of correct matches. In order to reflect the information on the overall cognition, after the standardization of the three cognitive scores, the average value was calculated as the composite z-score, and the influence of AHEI on global cognition was analyzed.

### 2.4. Depressive Symptoms

A patient health questionnaire, a nine-item depression screening tool, was used to determine the frequency of depressive symptoms in the past 2 weeks. The answer categories of the nine items “none at all”, “a few days”, “more than half of the days”, and “almost every day” were given a score ranging from 0 to 3. The total score is the sum of the scores of nine items. The total score ranges from 0 to 27.

### 2.5. Covariates

Age, gender (male or female), race (Mexican American, other Hispanic, non-Hispanic White, non-Hispanic Black, other), education status (less than high school, high school, and higher than high school), marital status (married, unmarried, divorced, other), smoking status (never smoked, used to smoke, current smoke), alcohol drinking status (yes or no). Body mass index (BMI), diabetes, heart disease, and stroke. BMI was defined as weight/height^2^ (Kg/m^2^), and according to WHO criteria [20], participants were classified as underweight (≤18.5 Kg/m^2^), normal weight (18.5–24.9 Kg/m^2^), or overweight (≥25 Kg/m^2^). Diabetes, hypertension, heart disease, and stroke were defined based on self-reported status from diagnosis by doctors and were defined as yes or no.

### 2.6. Statistical Analysis

All data were analyzed by Stata15.0 (Stata Corporation, College Station, TX, USA) software version. Considering the complex sampling design of NHANES, a new sample weight (original 2-year sample weight divided by 2) was created according to the analysis guidelines of NHANES.

The basic characteristics of continuous variables were described by mean and standard deviation, while classified variables were described by percentage. Kruskal–Wallis test or one-way analysis of variance (ANOVA) was used to analyze the difference between continuous variables. The Chi-square test was used to analyze the differences between classified variables.

**Figure 2:**
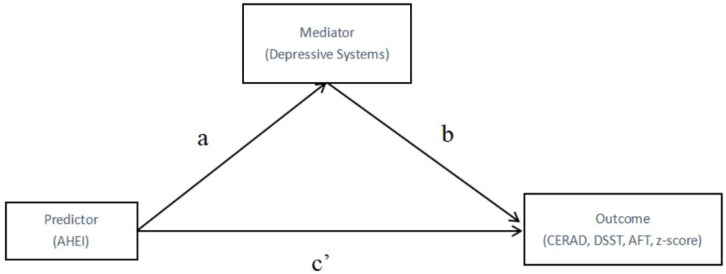
Path diagram of mediation model. (**a**): the effect of AHEI on the depressive systems; (**b**): the effect of depressive systems on cognitive function (CERAD, DSST, AFT, z-score); (**c’**): the direct effect of predictor on cognitive function (CERAD, DSST, AFT, z-score).

The weighted multiple linear regression was used to evaluate the relationship between AHEI-2010 and cognitive function. A linear regression model based on Karlson–Holm–Breen (KHB) method [21] was used to test the mediating role of depressive symptoms in the relationship between AHEI-2010 and cognitive function. Figure 2 provides a path model that indicates the mediation effect of the depressive symptoms. All reported *p-*value were two-sided; *p* < 0.05 was considered statistically significant.

## 3. Results

Table 1 presents the population characteristics according to the AHEI-2010 quartile. A total of 2644 participants were included in the analysis, with an average age of 69.43 years old, and the average score of AHEI-2010 was 47.06. Compared with the lowest quartile of AHEI-2010, the highest quartile was more likely to be male, non-Hispanic white, married, had smoked, and had a higher level of education (*p* < 0.001). With the increase in the AHEI-2010 quartile, the number of people drinking alcohol and suffering from hypertension increased, but the number of strokes decreased. In addition, the DSST and AFT scores of the highest AHEI-2010 quartile were significantly higher than those of the lowest quartile. There was no significant difference in age, BMI, and heart disease.

Table 2 showed the associations of AHEI-2010 and depressive symptoms with cognitive function measures. In the crude model, the highest quartile of AHEI-2010 was significantly correlated with DSST (β: 3.38; 95%CI: 1.61, 5.15) and comprehensive z-scores (β: 0.14; 95%CI: 0.04, 0.23). The higher quartile of AHEI-2010 were positively correlated with AFT. In addition, there was evidence of a linear trend in all the above three cognitive function measures. The scores of DSST, AFT, and comprehensive z-scores were significantly increased in the second to fourth quartile of AHEI-2010 (*p* trend < 0.01). In the fully adjusted model, the highest quartile was still significantly correlated with DSST, AFT, and comprehensive z-scores. In addition, the linear trend of AHEI-2010 in DSST, AFT, and comprehensive z-scores still existed (*p* trend < 0.01). Depressive symptoms were significantly negatively correlated with all cognitive function measures, and no significant correlation between AHEI-2010 and CERAD was found in all models.

Table 3 presented that higher AHEI scores were associated with lower depressive symptoms. Compared with the lowest quartile, the highest quartile group showed a significant negative correlation with depressive symptoms (β: −0.80; 95%CI: −1.24, −0.35). After adjusting for confounding factors, the results were generally consistent.

Table 4 presents the results of the relationship between AHEI-2010 components and cognitive functions. The intake of vegetables, nuts, and legumes was positively correlated with CERAD, DSST, AFT, and comprehensive z-score. Meanwhile, the intake of whole fruit was also associated with higher DSST scores and comprehensive z-scores. Additionally, intake of omega-3 fatty acids was significantly positively correlated with DSST and AFT. There was a significantly negative correlation between sodium intake and sugary beverage intake with CERAD. In addition, sodium intake was negatively correlated with comprehensive z-score. No significant correlations were found between whole grains, red meat and processed meat, polyunsaturated fatty acids, alcohol consumption, and any cognitive function measures.

The results of the mediation analysis are shown in Table 5. After adjusting for the confounding factors, the mediation effect of depressive symptoms was found in all four cognitive function measures. The total effect of AHEI-2010 on CERAD was 0.0255 (95%CI: 0.0008, 0.0503), the indirect effect of AHEI-2010 through depressive symptoms was 0.0036 (95%CI: 0.0009, 0.0064), and the proportion of mediating effect was 14.14%. Similarly, the mediation proportion of AHEI-2010 in DSST, AFT, and comprehensive z-score was 9.10%, 9.15%, and 10.47%, respectively.

## 4. Discussion

This cross-sectional study used the NHANES data from 2011–2012 and 2013–2014 to analyze the relationship between AHEI-2010 and cognitive function and the mediating role of depressive symptoms and found that higher dietary quality was associated with better cognitive function among older adults, especially in terms of processing speed and executive function. In addition, depressive symptoms play a partial mediating role in the relationship between AHEI-2010 and cognitive function.

Our findings on the relationship between AHEI-2010 and cognitive function are consistent with previous studies. For example, in two international parallel trials, ONTARGET (Ongoing Telmisartan Alone and in Combination with Ramipril Global Endpoint Trial) and TRANSCEND (Telmisartan Randomized Assessment Study in ACE Intolerant Subjects with Cardiovascular Disease), 27,860 participants were followed up for 56 months and found that higher dietary quality was associated with a reduced risk of cognitive decline [22]. A follow-up study of Chinese adults found that sticking to a healthy diet in middle age was associated with a reduced risk of cognitive impairment in old age [10]. Another survey of 806 participants from the Sguimiento University Navarra (SUN) college graduate cohort found a beneficial association between Mediterranean-Dash Intervention for Neurodegenerative Delay (MIND) Diet and AHEI-2010 diet patterns and cognitive function [23].

The effect of diet quality on cognitive function may be partly mediated by depression. We found that the AHEI-2010 score was significantly negatively associated with depressive symptoms, and so was the relationship between depressive symptoms and cognitive function. In the analysis of AHEI-2010 components, we found that the intake of vegetables, fruits and nuts, and legumes recommended by AHEI-2010 was associated with better cognitive function, while the intake of sugary drinks and sodium was the opposite. At present, there is evidence that AHEI-2010 restricted components, especially the intake of sugary drinks, red meat, and processed foods, which might increase the risk of body inflammation and change intestinal microflora. In turn, they might have negative effects on the brain and increase the risk of developing depression [13,24,25,26]. In addition, previous studies have shown that higher dietary sodium intake increases the risk of hypertension, which is an important risk factor for depression. Therefore, long-term high sodium intake may affect neurological function over time [27,28,29]. Dash et al. have reviewed animal experiments and shown that high sodium, saturated fat, and added sugar have negative effects on brain function through damage to frontal, limbic, and hippocampal areas of the brain [30,31], while the increased intake of fruits and vegetables was associated with a lower risk of depression in adults [32,33,34]. The reasons for the link between fruit and vegetable intake and depression may be as follows. Firstly, the imbalance between the production of free radicals and antioxidation is thought to be the cause of depression [35,36,37]. Vegetables and fruits contain polyphenols and nutrients with anti-inflammatory properties such as β-carotene, vitamin E, and vitamin C [38]. Secondly, deficiency of folic acid or vitamin B12 increases homocysteine levels and increases the risk of depression [39]. In addition, legumes are rich in tryptophan [40]. Tryptophan can synthesize the neurotransmitter serotonin, and sufficient serotonin can prevent brain damage during aging [41].

Meanwhile, some cross-lagged analyses in older adults have shown that depressive symptoms may be a risk factor for cognitive decline [15,16,17]. These are consistent with our findings. Depression may be associated with a cognitive impairment through a variety of mechanisms, including loss of hippocampal volume caused by excessive secretion of corticosteroids, resulting in neurotoxicity; inhibition of hippocampal neurogenesis [42,43]; increased deposition of b-amyloid plaques; and lack of nerve growth factors such as brain-derived neurotrophic factor affect cognitive function [44].

Based on the above evidence, it can be seen that diet quality may affect cognitive function through depression symptoms. Therefore, active dietary intervention in people with depressive symptoms in later life may help maintain better cognitive function and delay the onset of dementia. Even a small reduction in the risk of dementia could have large health benefits for the public due to the predicted rapid increase in the number of people with dementia in the future.

Our study has several advantages. To our knowledge, this is the first time to explore the mediating effect of depressive symptoms on the association between dietary quality and cognitive function. Second, the sample size is large enough, and because the quality of the NHANES survey is very high, the representativeness of the sample is pretty good, which can be extended to the whole US population. However, the disadvantages of the article should not be ignored. First of all, this is a cross-sectional study in which causality cannot be determined. Second, there may be recall bias in the process of self-reported dietary data collection.

## 5. Conclusions

In conclusion, the results of this study suggest that a better quality of diet may reduce the degree of depressive symptoms and thus maintain better cognitive function. It is worth noting that dietary quality is an important and effective means of intervention, which is of great significance for the prevention of age-related cognitive decline and even dementia.

## Figures and Tables

**Table 1:** Basic characteristics of the included sample (*n* = 2644).

Characteristics	All Participants	Quartile of AHEI-2010				
		Q1 (0–40.04)	Q2 (40.04–46.46)	Q3 (46.46–53.64)	Q4 (53.64–100)	*p-*Value
No. of participants	2644	661	661	661	661	
Age, mean (SD) ^a^	69.43 (6.77)	69.41 (6.81)	69.60 (7.01)	69.38 (6.80)	69.34 (6.46)	0.907
Gender, *n* (%) ^b^						<0.001
Male	1321 (49.96)	137 (20.73)	324 (49.02)	386 (58.40)	474 (71.71)	
Female	1323 (50.04)	524 (79.27)	337 (50.98)	275 (41.60)	187 (28.29)	
Race, *n* (%) ^b^						<0001
Mexican American	221 (8.36)	43 (6.51)	58 (8.77)	58 (8.77)	62 (9.38)	
Other Hispanic	258 (9.76)	59 (8.93)	63 (9.53)	70 (10.59)	66 (9.98)	
Non-Hispanic White	1309 (49.51)	320 (48.41)	336 (50.83)	332 (50.23)	321 (48.56)	
Non-Hispanic Black	629 (23.79)	204 (30.86)	165 (24.96)	137 (20.73)	123 (18.61)	
Other	227 (8.59)	35 (5.30)	39 (5.90)	64 (9.68)	89 (13.46)	
Marital status, *n* (%) ^b^						<0.001
Marry	1470 (55.60)	315 (47.66)	328 (49.62)	402 (60.82)	425 (64.30)	
Widowed	499 (18.87)	161 (24.36)	133 (20.12)	111 (16.79)	94 (14.22)	
Divorced	386 (14.60)	106 (16.04)	125 (18.91)	82 (12.41)	73 (11.04)	
Other	289 (10.93)	79 (11.95)	75 (11.35)	66 (9.98)	69 (10.44)	
BMI status, *n* (%) ^b^						0.104
<18.5	37 (1.40)	13 (1.97)	11 (1.66)	8 (1.21)	5 (0.76)	
18.5–25	776 (29.35)	201 (30.41)	169 (25.57)	196 (29.65)	210 (31.77)	
>25	1831 (69.25)	447 (67.62)	481 (72.77)	457 (69.14)	446 (67.47)	
Education, *n* (%) ^b^						<0.001
<High school	647 (24.47)	164 (24.81)	192 (29.05)	155 (23.45)	136 (20.57)	
High school	625 (23.64)	195 (29.50)	153 (23.15)	147 (22.24)	130 (19.67)	
>High school	1372 (51.89)	302 (45.69)	316 (47.81)	359 (54.31)	395 (59.76)	
Smoking status, *n* (%) ^b^						<0.001
Nonsmokers	1280 (48.41)	366 (55.37)	321 (48.56)	307 (46.44)	286 (43.27)	
Ex-smokers	1028 (38.88)	202 (30.56)	243 (36.76)	265 (40.09)	318 (48.11)	
Current smokers	334 (12.63)	92 (13.92)	96 (14.52)	89 (13.46)	57 (8.62)	
Alcohol drinking, n (%) ^b^	1818 (68.76)	390 (59.00)	450 (68.08)	471 (71.26)	507 (76.60)	<0.001
Diabetes, n (%) ^b^	121 (4.58)	28 (4.24)	32 (4.84)	26 (3.93)	35 (5.30)	0.639
Heart attack, *n* (%) ^b^	236 (8.93)	48 (7.26)	75 (11.35)	56 (8.47)	57 (8.62)	0.064
Stroke, *n* (%) ^b^	186 (7.03)	53 (8.02)	62 (9.38)	34 (5.14)	37 (5.60)	0.007
Hypertension, *n* (%) ^b^	751 (28.40)	162 (24.51)	177 (26.78)	203 (30.71)	209 (31.61)	0.012
CERAD, mean (SD) ^c^	24.98 (6.49)	25.49 (6.46)	24.70 (6.64)	24.74 (6.57)	25.01 (6.26)	0.098
DSST, mean (SD) ^c^	46.31 (17.13)	45.38 (17.36)	45.37 (17.31)	46.24 (17.06)	48.24 (16.67)	0.006
AFT, mean (SD) ^c^	16.72 (5.47)	16.10 (5.30)	16.67 (5.61)	16.79 (5.41)	17.32 (5.51)	0.001
Z-score, mean (SD) ^c^	0.02 (0.78)	0.01 (0.77)	−0.02 (0.83)	0.01 (0.79)	0.08 (0.73)	0.107

^a^ *p*-value was tested by Kruskal–Wallis test; ^b^ *p*-value was tested by Chi-square test; ^c^ *p*-value was tested by one-way analysis of variance (ANOVA).

**Table 2:** Associations of quartile of AHEI-2010 and depressive symptoms with cognitive function: NHANES: 2011–2014.

	CERAD ^c^	*p*-Value	DSST ^d^	*p*-Value	AFT ^e^	*p*-Value	Z-score ^f^	*p*-Value
	β (95% CI)		β (95% CI)		β (95% CI)		β (95% CI)	
Crude model ^a^								
Depressive symptoms	−0.12(−0.22, −0.03)	**0.011**	−0.70 (−0.94, −0.46)	**<0.001**	−0.16 (−0.23, −0.10)	**<0.011**	−0.03 (−0.04, −0.02)	**<0.001**
Quartile of AHEI-2010								
Q1(0–40.04)	0.00 (Ref)		0.00 (Ref)		0.00 (Ref)		0.00 (Ref)	
Q2(40.04–46.46)	−0.18 (−1.09, 0.74)	0.697	1.47 (−0.72, 3.67)	0.182	1.05 (0.07, 2.04)	**0.037**	0.06 (−0.07, 0.18)	0.38
Q3(46.46–53.64)	−0.50 (−1.35, 0.36)	0.246	1.51 (−0.36, 3.39)	0.11	0.85 (0.12, 1.57)	**0.023**	0.02 (−0.07, 0.11)	0.596
Q4(53.64–100)	0.03 (−0.77, 0.83)	0.942	3.38 (1.61, 5.15)	**0.001**	1.83 (0.92, 2.74)	**0.001**	0.14 (0.04, 0.23)	**0.006**
*p* trend		0.809		**0.001**		**<0.011**		**0.004**
Adjusted model ^b^								
Depressive symptoms	−0.11 (−0.20, −0.03)	**0.012**	−0.53 (−0.73, −0.33)	**<0.001**	−0.11 (−0.18, −0.05)	**0.001**	−0.02 (−0.03, −0.01)	**<0.001**
Quartile of AHEI-2010								
Q1 (0–40.04)	0.00 (Ref)		0.00 (Ref)		0.00 (Ref)		0.00 (Ref)	
Q2 (40.04–46.46)	0.31 (−0.61, 1.24)	0.495	2.25 (0.61, 3.90)	**0.009**	0.82 (−0.05, 1.69)	0.063	0.09 (−0.02, 0.20)	0.115
Q3 (46.46–53.64)	−0.04 (−0.99, 0.91)	0.929	1.52 (−0.27, 3.31)	0.093	0.36 (−0.30, 1.01)	0.277	0.03 (−0.05, 0.12)	0.44
Q4 (53.64–100)	0.60 (−0.27, 1.47)	0.169	3.37 (2.03, 4.71)	**0.001**	1.14 (0.25, 2.04)	**0.014**	0.14 (0.06, 0.23)	**0.002**
*p* trend		0.054		**<0.001**		**0.011**		**<0.001**

^a^ Crude model did not adjust for any confounding factors. ^b^ Adjusted model adjusted for age, gender, education, marital status, race, smoke status, alcohol drinking status, BMI, stroke, heart attack, diabetes, and hypertension. ^c^ CERAD—Consortium to Establish a Registry for Alzheimer’s disease Word Learning sub-test; ^d^ DSST—Digit Symbol Substitution Test; ^e^ AFT—Animal Fluency Test; ^f^ Z-score was calculated by summing the z-scores (i.e., (individual value − mean value)/SD) for performance across the three cognitive tests (i.e., CERAD, DSST, AFT). Bold font indicates that *p*-value is statistically significant (*p* < 0.05).

**Table 3:** β (*p*) of the depressive symptoms according to the quartile of AHEI-2010.

	Depressive Symptoms			
	Crude Model ^a^		Adjusted Model ^b^	
	β (95% CI)	*p-*Value	β (95% CI)	*p*-Value
Quartile of AHEI-2010				
Q1 (0–40.04)	0.00 (Ref)		0.00 (Ref)	
Q2 (40.04–46.46)	−0.14 (−0.63, 0.34)	0.552	−0.2 (−0.73, 0.32)	0.438
Q3 (46.46–53.64)	−0.09 (−0.60, 0.41)	0.707	−0.46 (−1.02, 0.11)	0.109
Q4 (53.64–100)	−0.80 (−1.24, −0.35)	**0.001**	−1.27 (−1.80, −0.75)	**<0.001**

^a^ Crude model did not adjust any confounding factors. ^b^ Adjusted model adjusted for age, gender, education, marital status, race, smoke status, alcohol drinking status, BMI, stroke, heart attack, diabetes, and hypertension. Bold font indicates that *p*-value is statistically significant (*p* < 0.05).

**Table 4:** Associations of AHEI-2010 components with cognitive function: NHANES: 2011–2014(*n* = 2644).

	CERAD ^c^ β (95% CI)	*p-*Value	DSST ^d^ β (95% CI)	*p-*Value	AFT ^e^ β (95% CI)	*p*-Value	Z-Score ^f^ β (95% CI)	*p*-Value
Crude model ^a^								
Vegetables	0.44 (0.29, 0.58)	**<0.001**	1.13 (0.75, 1.51)	**<0.001**	0.48 (0.32, 0.63)	**<0.001**	0.07 (0.05, 0.08)	**<0.001**
Whole fruit	0.20 (0.03, 0.37)	**0.021**	0.94 (0.62, 1.27)	**<0.001**	0.15 (−0.004, 0.30)	0.056	0.03 (0.02, 0.05)	**0.001**
Whole grains	−0.62 (−0.94, −0.31)	**<0.001**	−0.84 (−1.50, −0.18)	**0.014**	0.25 (−0.14, 0.65)	0.204	−0.05 (−0.08, −0.01)	**0.012**
Sugar fruit juice	−0.08 (−0.15, −0.01)	**0.032**	−0.01 (−0.17, 0.14)	0.867	−0.05 (−0.13, 0.02)	0.142	−0.01 (−0.02, 0.001)	0.079
Nuts and legumes	0.18 (0.11, 0.26)	**<0.001**	0.51 (0.33, 0.69)	**<0.001**	0.19 (0.12, 0.26)	**<0.001**	0.03 (0.02, 0.04)	**<0.001**
Red meat	0.01 (−0.07, 0.08)	0.873	0.10 (−0.12, 0.32)	0.367	0.05 (−0.02, 0.12)	0.194	0.004 (−0.01, 0.01)	0.416
ω-3 fatty acids	0.20 (−0.70, 1.09)	0.657	1.35 (−0.33, 3.04)	0.112	0.60 (0.20, 1.00)	**0.004**	0.06 (−0.02, 0.14)	0.163
PUFA	−0.02 (−0.12, 0.08)	0.699	0.29 (0.10, 0.48)	**0.004**	0.09 (−0.01, 0.19)	0.083	0.01 (−0.005, 0.02)	0.245
Sodium	−0.17 (−0.38, 0.03)	0.088	−0.67 (−1.21, −0.12)	**0.019**	−0.45 (−0.68, −0.22)	**<0.001**	−0.04 (−0.07, −0.02)	**0.001**
Alcohol	−0.25 (−0.34, −0.17)	**<0.001**	−0.67 (−0.86, −0.48)	**<0.001**	0.001 (−0.08, 0.08)	0.985	−0.03 (−0.04, −0.02)	**<0.001**
Adjusted model ^b^								
Vegetables	0.34 (0.19, 0.49)	**<0.001**	0.52 (0.22, 0.83)	**0.001**	0.31 (0.19, 0.43)	**<0.001**	0.05 (0.03, 0.06)	**<0.001**
Whole fruit	0.13 (−0.02, 0.29)	0.084	0.59 (0.35, 0.82)	**<0.001**	0.10 (−0.04, 0.24)	0.167	0.02 (0.01, 0.04)	**0.007**
Whole grains	−0.13 (−0.43, 0.17)	0.387	−0.24 (−1.06, 0.59)	0.565	0.004 (−0.44, 0.45)	0.985	−0.01 (−0.05, 0.02)	0.413
Sugar fruit juice	−0.07 (−0.14, −0.01)	**0.046**	0.004 (−0.12, 0.13)	0.951	−0.05 (−0.11, 0.02)	0.141	−0.01 (−0.01, 0.001)	0.083
Nuts and legumes	0.15 (0.08, 0.21)	**<0.001**	0.35 (0.21, 0.50)	**<0.001**	0.14 (0.09, 0.20)	**<0.001**	0.02 (0.02, 0.03)	**<0.001**
Red meat	0.002 (−0.06, 0.07)	0.947	0.01 (−0.13, 0.015)	0.919	−0.01 (−0.08, 0.05)	0.672	−0.001 (−0.01, 0.01)	0.986
ω-3 fatty acids	0.23 (−0.55, 1.01)	0.551	1.21 (0.09, 2.34)	**0.036**	0.57 (0.27, 0.87)	**<0.001**	0.06 (−0.01, 0.12)	0.095
PUFA	−0.07 (−0.15, 0.008)	0.076	0.08 (−0.10, 0.26)	0.381	0.05 (−0.04, 0.14)	0.278	−0.001 (−0.01, 0.01)	0.766
Sodium	−0.25 (−0.49, −0.01)	**0.038**	−0.30 (−0.81, 0.20)	0.228	−0.16 (−0.37, 0.05)	0.136	−0.03 (−0.06, −0.02)	**0.036**
Alcohol	0.05 (−0.10, 0.20)	0.522	−0.19 (−0.49, 0.10)	0.198	−0.02 (−0.16, 0.12)	0.786	−0.001 (−0.02, 0.02)	0.873

^a^ Crude model did not adjust any confounding factors. ^b^ Adjusted model adjusted for age, gender, education, marital status, race, smoke status, alcohol drinking status, BMI, stroke, heart-attack, diabetes, and hypertension. ^c^ CERAD—Consortium to Establish a Registry for Alzheimer’s disease Word Learning sub-test; ^d^ DSST—Digit Symbol Substitution Test; ^e^ AFT—Animal Fluency Test; ^f^ Z-score was calculated by summing the z-scores (i.e., (individual value − mean value)/SD) for performance across the three cognitive tests (i.e., CERAD, DSST, AFT). Bold font indicates that *p*-value is statistically significant (*p* < 0.05).

**Table 5:** The mediating proportion of depressive symptoms on the association between AHEI-2010 and cognitive function.

	Direct Effect β (95%CI)	*p-* Value	Indirect Effect β (95%CI)	*p-* Value	Total Effect β (95%CI)	*p-* Value	Proporation Mediated (%)
CERAD ^a^	0.0219 (−0.0029, 0.0467)	0.083	0.0036 (0.0009, 0.0064)	0.043	0.0255 (0.0008, 0.0503)	0.010	14.14
DSST ^b^	0.1298 (0.0754, 0.1842)	<0.001	0.0130 (0.0041, 0.0219)	<0.001	0.1428 (0.0885, 0.1971)	0.004	9.1
AFT ^c^	0.0298 (0.0087, 0.0508)	0.006	0.0030 (0.0007, 0.0053)	0.002	0.0328 (0.0118, 0.0538)	0.010	9.15
Z-score ^d^	0.0049 (0.0023, 0.0076)	<0.001	0.0006 (0.0002, 0.0010)	<0.001	0.0055 (0.0028, 0.0082)	0.005	10.47

Adjusted for age, gender, education, marital status, race, smoke status, alcohol drinking status, BMI, stroke, heart attack, diabetes, and hypertension. ^a^ CERAD—Consortium to Establish a Registry for Alzheimer’s disease Word Learning sub-test; ^b^ DSST—Digit Symbol Substitution Test; ^c^ AFT—Animal Fluency Test; ^d^ Z-score was calculated by summing the z-scores (i.e., (individual value − mean value)/SD) for performance across the three cognitive tests (i.e., CERAD, DSST, AFT).

## Data Availability

The data are available at https://www.cdc.gov/nchs/nhanes/index.htm (access date: 11 July 2022).
